# Supplementary material for: Detection of Nitroaromatic Explosives in Air by Amino-Functionalized Carbon Nanotubes
Source: Nanomaterials (Basel). 2022 Apr 8;12(8):1278. doi: 10.3390/nano12081278 (PMC9027238; doi:10.3390/nano12081278)
Supplement: Supplementary file 1 [file nanomaterials-12-01278-s001.zip › nanomaterials-1627630-supplementary.pdf]

Supplemental Information

Detection of Nitroaromatic Explosives in Air by Amino-Functionalized Carbon Nanotubes

Claudio Ferrari [1,*], Giovanni Attolini [1], Matteo Bosi [1], Cesare Frigeri [1], Paola Frigeri [1], Enos Gombia [1], Laura Lazzarini [1], Francesca Rossi [1], Luca Seravalli [1], Giovanna Trevisi [1], Riccardo Lolli [1,2], Lucrezia Aversa [3], Roberto Verucchi [3], Nahida Musayeva [4], Muhammad Alizade [4], Sevinj Quluzade [4], Teimur Orujov [4], Francesco Sansone [5], Laura Baldini [5] and Francesco Rispoli [5]

1 IMEM-CNR Institute, Parco Area delle Scienze 37/A, 43124 Parma, Italy; giovanni.attolini@imem.cnr.it (G.A.); matteo.bosi@imem.cnr.it (M.B.); cesare.frigeri@imem.cnr.it (C.F.); paola.frigeri@imem.cnr.it (P.F.); enos.gombia@imem.cnr.it (E.G.); laura.lazzarini@imem.cnr.it (L.L.); francesca.rossi@imem.cnr.it (F.R.); luca.seravalli@imem.cnr.it (L.S.); giovanna.trevisi@imem.cnr.it (G.T.); rcrd.lolli@gmail.com (R.L.);

2 Department of Physics and Earth Science, Ferrara University, Via Saragat 1, 44122 Ferrara, Italy;

3 IMEM-CNR Institute, c/o FBK, Via alla Cascata 56/C, 38123 Povo-Trento, Italy; lucrezia.aversa@imem.cnr.it (L.A.); roberto.verucchi@imem.cnr.it (R.V.)

4 Institute of Physics, Azerbaijan National Academy of Sciences, H. Javid Avenue 131, Az-1143, Baku,, Azerbaijan; nahida@mail.ru (N.M.); mehemmedelizade97@gmail.com (M.A.); sgseva92@gmail.com (S.Q.); timphysics1@gmail.com (T.O.)

5 Department of Chemistry, Life Sciences and Environmental Sustainability, University of Parma, Parco Area delle Scienze, 17/a, 43124 Parma, Italy; francesco.sansone@unipr.it (F.S.); laura.baldini@unipr.it (L.B.); francesco.rispoli1@unipr.it (F.R.)

* Correspondence: claudio.ferrari@imem.cnr.it; Tel.: +39-0521269222

1) Functionalization of MWCNTs

COOH−MWCNT: MWCNTs (100 mg) were added to a round-bottomed flask containing 4 mL of HNO_3 (69%) and 12 mL of H_2SO_4 (95%). The mixture was sonicated for 5 h at room temperature, then cold water (200 mL) was added and the suspension was filtered through a Millipore membrane (JHWP, 0.45 µm). The black powder was washed with deionized water until neutralization, then with methanol and diethyl ether yielding 99 mg of oxidized MWCNTs. FT-IR: cm^{-1} 1729.5 (C=O), 1636.0 (C=C).

NH_2−C_2−MWCNT: COOH-MWCNTs (30 mg) were sonicated for 30 min in 15 mL of EDA, then 0.25 g of N,N′-dicyclohexylcarbodiimide (DCC) were added and the mixture was stirred at 70 °C for 72 h. The suspension was filtered under vacuum on a 0.45 µm PTFE Millipore membrane and the solid was washed on the filter with DMF (100 mL), methanol (100 mL) and diethyl ether (100 mL) obtaining 25 mg of NH_2-C_2−MWCNT. Elemental analysis: C 83.48%, H 0.92%, N 3.00%

*Corresponding author: Claudio.Ferrari@imem.cnr.it, IMEM-CNR Institute, Parco Area delle Scienze 37/A, Parma, Italy

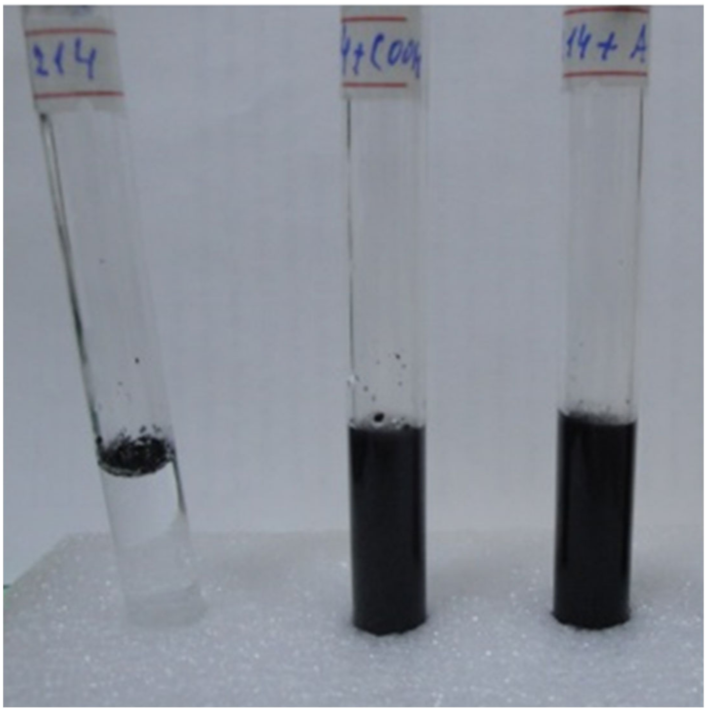

Figure S1: Dispersibility behavior in water of (from left to right): pristine MWCNTs, COOH−MWCNT and NH_2−C_2−MWCNT. The picture was taken four weeks after dispersion of the material in water by sonication.

1.1 Synthesis of NH_2−C_2−MWCNT

COOH−MWCNTs (30 mg) were sonicated for 30 min in 15 mL of EDA, then 0.25 g of N,N'−dicyclohexylcarbodiimide (DCC) were added and the mixture was stirred at 70 °C for 72 h. The suspension was filtered under vacuum on a 0.45 µm PTFE Millipore membrane and the solid was washed on the filter with DMF (100 mL), methanol (100 mL) and diethyl ether (100 mL) obtaining 25 mg of NH_2−C_2−MWCNT. Elemental analysis: C 83.48%, H 0.92%, N 3.00%

1.2 Synthesis of NH_2−C_6−MWCNT

30 mg of COOH−MWCNT were dispersed by sonication (30 min) in 10 mL of dry DMF. 0.2 g of 1.6-hexamethilendiamine and 0,15 g of N,N'−dicyclohexylcarbodiimide were added and the mixture was heated to 70°C under magnetic stirring for 72 h. The suspension was then filtered under vacuum on a 0.45 µm Millipore membrane in PTFE and the residue was washed with DMF (150 mL), methanol (150 mL) and diethyl ether (200 mL), affording 25 mg of NH_2−C_6−MWCNTs.

*Corresponding author: Claudio.Ferrari@imem.cnr.it, IMEM-CNR Institute, Parco Area delle Scienze 37/A, Parma, Italy

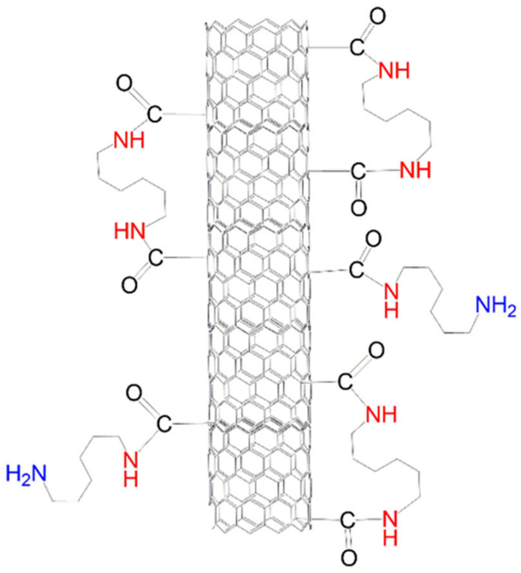

)

Figure S2: Schematic representation of the additional amide groups produced on the surface of NH2−C_6−MWCNT by the reaction between the terminal amine units of some linked chains with other COOH on the same MWCNT

2) XPS analysis

X-ray photoelectron spectroscopy (XPS) has been carried out in an Ul-tra-High-Vacuum (UHV) system, equipped with a non-monochromatized X-ray source (Mg Kα photons at 1253.6 eV) and a VSW HA100 hemispherical analyzer, leading to a total energy resolution of 0.86 eV. The binding energy (BE) scale of XPS spectra was calibrated by using the Au 4f peak at 84.0 eV as a reference. The core level analysis has been performed by Voigt line-shape deconvolution after background subtraction of a Shirley function.

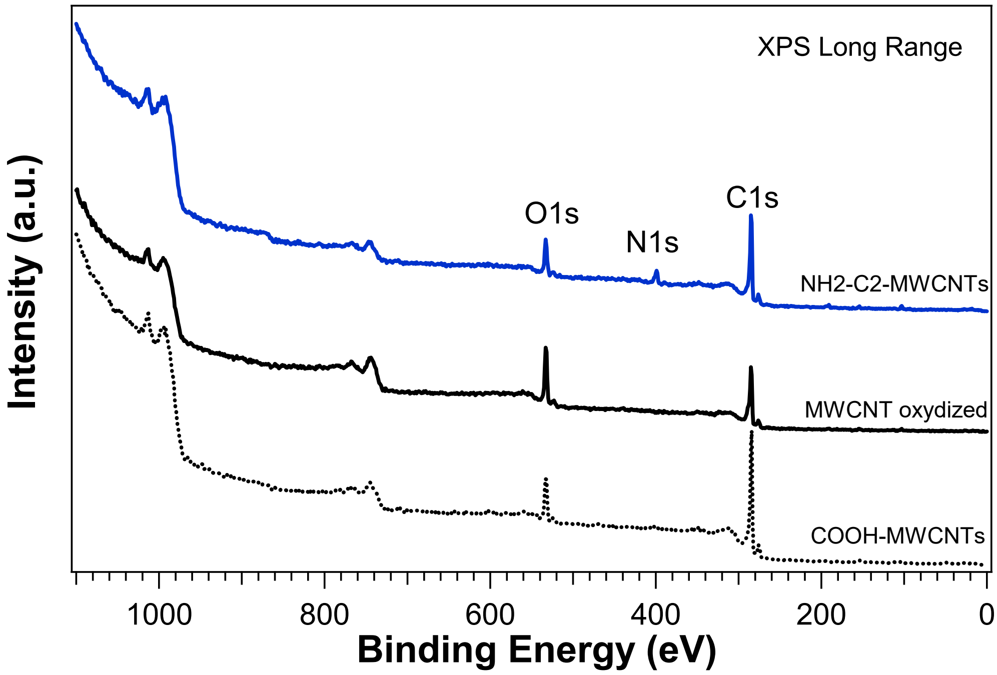

Figure S3: XPS spectra of pristine MWCNT, COOH−MWCNT and NH_2−C_2−MWCNT. Main core levels are labelled, large bands in the 700-1100 energy range are Auger peaks.

*Corresponding author: Claudio.Ferrari@imem.cnr.it, IMEM-CNR Institute, Parco Area delle Scienze 37/A, Parma, Italy

Table S1: XPS line shape analysis of C1s, O1s and N1s core level, showing peak assignment, energy position (Binding Energy, BE) and area percentage for different MWCNTs

Core level components		Pristine CNTs %	COOH-MWCNTs %	NH_2–C_2–MWCNTs %	NH_2–C_6–MWCNTs %
C1s	C=C (~284.5eV)	83.0	62.8	73.3	52.6
	C–C (~285.5eV)	6.8	15.1	11.2	23.7
	C–O/C–N (~286.6eV)	5.1	8.5	8.9	16.3
	C=O/CON (~288.1eV)	1.0	2.6	1.9	4.5
	COOH (~289.1)	1.5	7.5	1.5	2.5
	Shake-up (~290.8)	2.6	3.6	3.2	0.4
O1s	C–O (~533.5eV)	55.2	68.4	47.5	43.0
	C=O (~531eV)	44.8	31.6	52.5	57.0
N1s	NH_2 amine (~399.5eV)	-	-	50.1	20.2
	CON amide (~400.5eV)	-	-	49.9	79.8

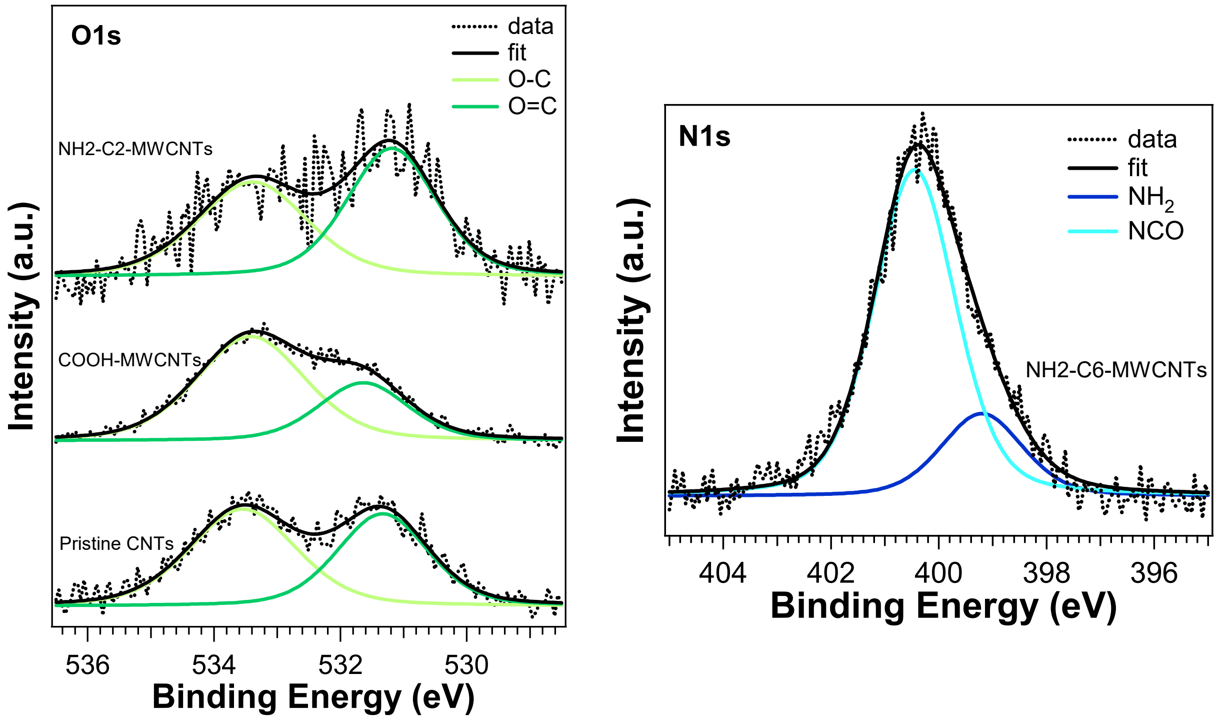

Figure S4: Left: XPS O1s core level spectra of Pristine MWCNTS, COOH–MWCNTs, and NH_2–C_2–MWCNTs; normalized to the peaks' height. Right: N1s core level spectra of NH_2–C_6–MWCNTs.

Lineshape analysis of O1s core level shows presence of C–O and C=O groups located at about 531 and 533.5eV, with specific weight varying due to the different chemical treatment. All spectra are normalized to peak height and background subtracted. XPS characterization of NH_2–C_6–MWCNTs N1s core level shows a modified ratio between amino NH_2 and amide CON groups. A 80% weight of amide groups is measured compared to 50% detected in NH_2–C_2–MWCNTs.

3) Dependence of sensor sensitivity on the degree of functionalization

The sensitivity of two sensors obtained from MWCNTs with an increasing degree of functionalization was tested with acetic acid (AA) and ammonia (NH_3), just exposing the sensor to a cotton swab impregnated by these

*Corresponding author: Claudio.Ferrari@imem.cnr.it, IMEM-CNR Institute, Parco Area delle Scienze 37/A, Parma, Italy

substances. The comparison is not quantitative, but the experiment confirms the dependence of sensitivity on the degree of functionalization. Previous tests performed on sensor realized with pristine MWCNTs did not show any reaction to the exposure to such substances.

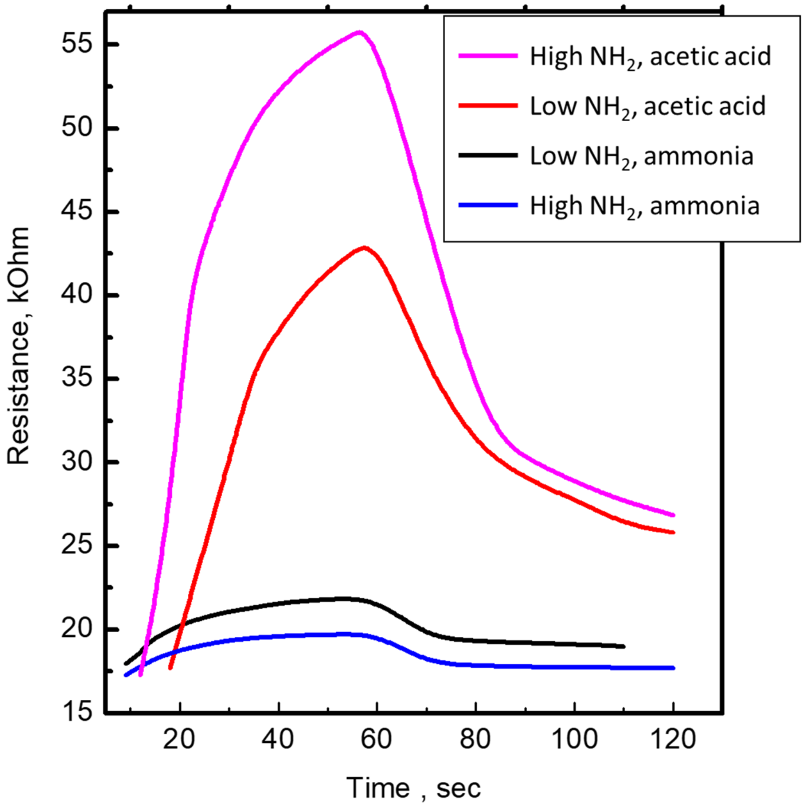

Figure S5: Resistance change under ammonia and acetic acid exposure in two sensors based on two different batches of NH_2–C_2–MWCNTs having a different degree of functionalization. The black and red curves are relative to the least functionalized sensor, while the blue and pink curves to the sensor realized from the batch of CNTs having the highest density of NH_2 groups. The analyte for the pink and red curves is acetic acid, for the black and blue curves is ammonia.

*Corresponding author: Claudio.Ferrari@imem.cnr.it, IMEM-CNR Institute, Parco Area delle Scienze 37/A, Parma, Italy
